# Correction to “Expression of Dehydroshikimate Dehydratase in Sorghum Improves Biomass Yield, Accumulation of Protocatechuate, and Biorefinery Economics”

**DOI:** 10.1021/acssuschemeng.2c05876

**Published:** 2022-10-20

**Authors:** Yang Tian, Minliang Yang, Chien-Yuan Lin, Joon-Hyun Park, Chuan-Yin Wu, Ramu Kakumanu, Christopher M. De Ben, Jutta Dalton, Khanh M. Vuu, Patrick M. Shih, Edward E. K. Baidoo, Stephen Temple, Daniel H. Putnam, Henrik V. Scheller, Corinne D. Scown, Aymerick Eudes

In our original article (https://pubs.acs.org/doi/full/10.1021/acssuschemeng.2c01160), it has come to our attention that the images for Figure 3 and Figure 4 have been interchanged during production of the article. The correct images are shown below.

**Figure 3:**
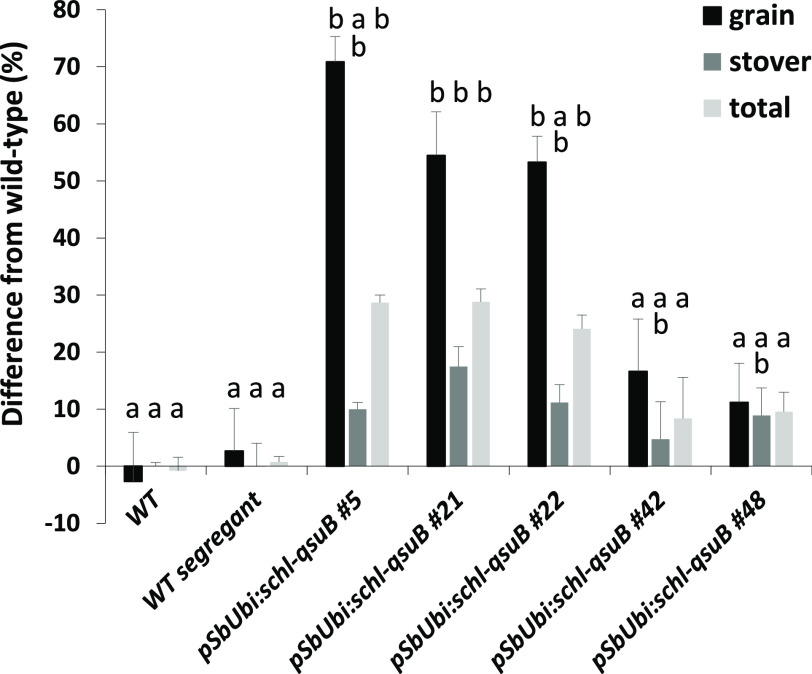
Biomass yields from WT and QsuB sorghum lines grown in the field. Values are means ± SE of four biological replicates (*n* = 4 plots). Columns with the same letter indicate lines that were not different (multivariate ANOVA and Duncan’s test for multiple comparisons, *P* < 0.05). The letters indicate comparison within each group (grain, stover, total) and not between these groups.

**Figure 4:**
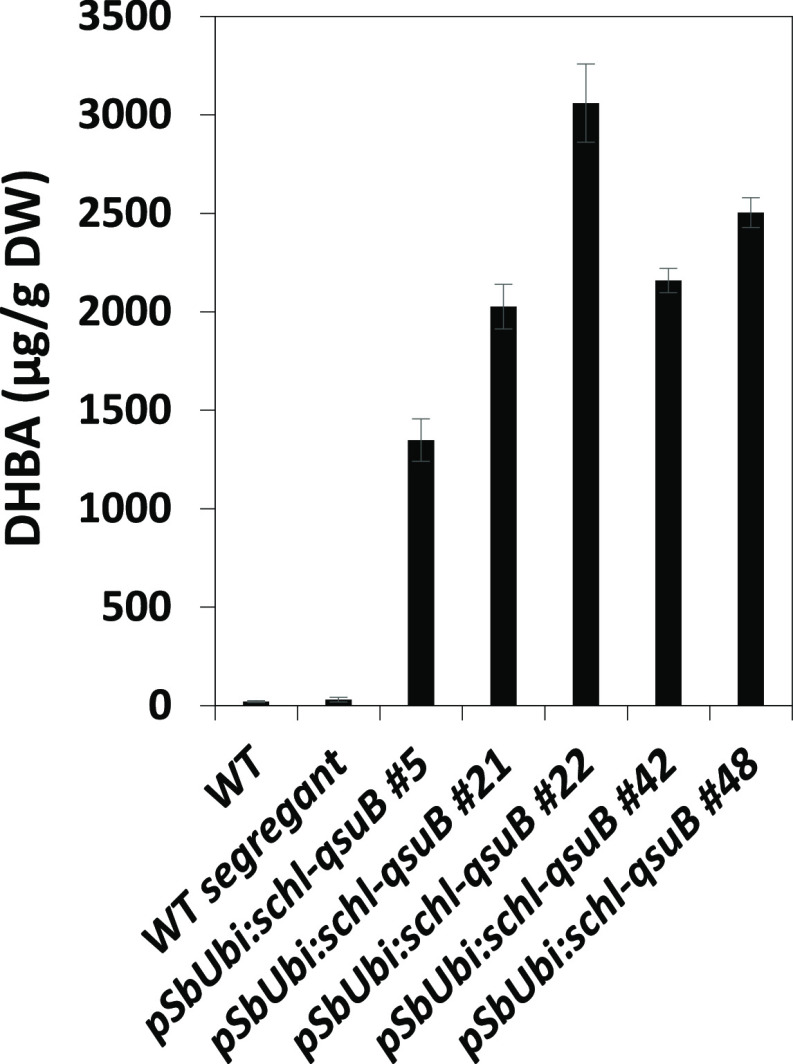
DHBA content in stover biomass from WT and QsuB sorghum lines grown in the field. Values are means ± SD of four biological replicates (*n* = 4 plots).

